# Proteomics and bioinformatics analysis of follicular fluid from patients with polycystic ovary syndrome

**DOI:** 10.3389/fmolb.2022.956406

**Published:** 2022-08-22

**Authors:** Wenqi Wang, Qi Jiang, Yue Niu, Qiaoqiao Ding, Xiao Yang, Yanjun Zheng, Jing Hao, Daimin Wei

**Affiliations:** ^1^ Center for Reproductive Medicine, Shandong University, Jinan, China; ^2^ Medical Integration and Practice Center, Shandong University, Jinan, China; ^3^ Key Laboratory of Reproductive Endocrinology of Ministry of Education, Shandong University, Jinan, China; ^4^ Shandong Key Laboratory of Reproductive Medicine, Jinan, China; ^5^ Key Laboratory of The Ministry of Education for Experimental Teratology, Department of Histology and Embryology, School of Basic Medicine Sciences, Cheeloo College of Medicine, Shandong University, Jinan, China

**Keywords:** polycystic ovary syndrome, follicular fluid, proteomics, tandem mass tag, ELISA

## Abstract

**Objectives:** Polycystic ovary syndrome (PCOS) is a common endocrine and metabolic disorder with heterogeneous manifestations and complex etiology. We used quantitative proteomics analysis based on mass spectrometry to identify the differences in proteomics profiles for follicular fluid obtained from patients with or without PCOS and explore possible mechanisms underlying PCOS.

**Methods:** Follicular fluid samples were collected from infertile patients with (n = 9) or without (n = 9) PCOS. Total protein was extracted, quantitatively labeled with a tandem mass tag (TMT), and analyzed using liquid chromatography-mass spectrometry (LC‐MS). TMT-based proteomics and bioinformatics analysis were used to determine the differentially expressed proteins (DEPs) and understand the protein networks. The analysis included protein annotation, unsupervised hierarchical clustering, functional classification, functional enrichment and clustering, and protein-protein interaction analysis. Selected DEPs were confirmed by ELISA, and correlation analysis was performed between these DEPs and the clinical characteristics.

**Results:** In this study, we have identified 1,216 proteins, including 70 DEPs (32 upregulated proteins, 38 downregulated proteins). Bioinformatics analysis revealed that the inflammatory response, complement and coagulation cascades, activation of the immune response, lipid transport, and regulation of protein metabolic processes were co-enriched in patients with PCOS. Based on ELISA results, insulin-like growth factor binding protein 1 (IGFBP1) and apolipoprotein C2 (APOC2) were differentially expressed between patients with and without PCOS. Follicular IGFBP1 showed a positive correlation with the serum levels of high-density lipoprotein cholesterol (HDL-C) (r = 0.3046, *p* = 0.0419), but negatively correlated with the serum levels of anti-Müllerian hormone (AMH) (r = –0.2924, *p* = 0.0354) and triglycerides (r = –0.3177, *p* = 0.0246). Follicular APOC2 was negatively correlated with the serum apolipoprotein A1 (APOA1) levels (r = 0.4509, *p* = 0.0002).

**Conclusion:** Our study identified DEPs in the follicular fluid of patients with PCOS. Inflammatory response, complement and coagulation cascades, activation of the immune response, lipid transport, and regulation of protein metabolic process were deregulated in PCOS, which may play essential roles in the pathogenesis of PCOS.

## Introduction

Polycystic ovary syndrome (PCOS) is a common disorder encompassing reproductive, metabolic, and endocrine abnormalities, affecting 5–10% of women of reproductive age (Azziz et al., 2016; Alexiou et al., 2017). PCOS is characterized by oligo-ovulation or anovulation, hyperandrogenism, and polycystic ovarian morphology (Azziz et al., 2016; Hoeger et al., 2021), with common comorbidities of abnormal glucose and lipid metabolism, obesity, insulin resistance, hepatic steatosis, and a higher risk of type 2 diabetes and cardiovascular disease (Teede et al., 2010; Escobar-Morreale, 2018; Patel, 2018). Due to the high heterogeneity and complexity of PCOS, the pathogenesis and molecular basis of PCOS are not fully understood.

Follicular fluid (FF) exists in the antrum of the growing follicle, which is a transudate of plasma components and the secretions from granulosa and theca cells. It is mainly composed of proteins, steroids, metabolites, and polysaccharides, providing an essential microenvironment for oocyte growth, maturation, and ovulation (Rodgers and Irving-Rodgers, 2010). Therefore, alterations in the FF composition may reflect metabolism and the secretory activities of follicular cells, affect follicular development and oocyte quality, and help decipher the underlying pathophysiology of PCOS (Niu et al., 2017; Da Broi et al., 2018).

Proteomics analysis is a powerful approach to understanding complex biological processes (Zhang et al., 2014) and may contribute to a better understanding of the physiopathology of PCOS. Several studies have been performed using two-dimensional gel electrophoresis (2-DE) to analyze the FF of patients with PCOS. However, few differentially expressed proteins (DEPs) have been identified, with only six and seven DEPs detected in two recent studies (Kim et al., 2013; Sim et al., 2021). Improvements in proteomics technology, such as isobaric mass tagging through mass spectrometry (MS) with tandem mass tag (TMT) and isobaric tags for relative and absolute quantification (iTRAQ), have addressed this limitation by increasing the number of identified proteins within a limited sample size (Wiese et al., 2007). Isobaric mass tagging has several advantages, including high reproducibility, sensitivity, and sample multiplexing (Zhang et al., 2014). One study has used iTRAQ-based analysis to compare the protein profiles of FF from Indian PCOS patients and controls. The results suggested that proteins involved in extracellular matrix remodeling, the complement coagulation cascade, fibrinolysis, vasculature development, angiogenesis, lipid transport, and metabolism were deregulated in PCOS (Ambekar et al., 2015). Another TMT-based proteomic analysis study identified 41 DEPs between overweight or obese PCOS patients vs non-PCOS women and 19 DEPs between normal-weight PCOS patients vs non-PCOS women (Zhang et al., 2019). Although novel proteins and pathways potentially involved in the pathogenesis of PCOS have been found in recent years, it is still a challenge to identify the proteins that may contribute to the risk of developing PCOS. Only a small proportion of the proteome from the FF of patients with PCOS has been revealed. In the present study, we performed TMT-based liquid chromatography-tandem mass spectrometry (LC-MS/MS) technology and bioinformatics analysis to further investigate the proteomic changes in the FF from Chinese Han patients with or without PCOS (n = 9 per group) to identify additional differential proteins that may be associated with the pathogenesis of PCOS. Furthermore, we performed ELISA to validate two of the DEPs to confirm the reliability of this study. We also performed a correlation analysis between these DEPs and the clinical characteristics to better understand the pathogenesis of PCOS.

## Materials and methods

### Subjects

This study consisted of patients who underwent *in vitro* fertilization (IVF) or intracytoplasmic sperm injection (ICSI) in the Reproductive Medical Center, Shandong University, from August 2020 to February 2021. The Ethics Committee of Reproductive Medicine of Shandong University approved this study. All subjects gave written informed consent before participation. We used the Rotterdam criteria from 2003 to diagnose PCOS, which required at least two of the following three conditions and excluded other causes of hyperandrogenism and ovulation dysfunction: clinical and/or biochemical evidence of hyperandrogenism, oligo- and/or anovulation, and polycystic ovarian morphology (The Rotterdam ESHRE/ASRM‐sponsored PCOS consensus workshop group, 2004). The control group consisted of infertile women undergoing IVF due to tubal or male factors that met the following inclusion criteria: normal ovarian reserve (regular menstrual cycles, follicle-stimulating hormone (FSH) < 10 IU/L, and anti-Müllerian hormone (AMH) ≥ 1.5 ng/ml), no signs of hyperandrogenism, no endocrine diseases, and normal ovarian and uterine morphology confirmed by ultrasound. All recruited women were ≤40 years old and had a body mass index (BMI) ranging from 18 to 30 kg/m^2^. Women with a history of ovarian surgery, uterine malformations, thyroid disease, and chromosomal abnormalities were excluded. Baseline hormones were detected on days 2–5 of the menstrual cycle in all recruited subjects. For patients with PCOS in oligo- or anovulation, hormone levels were measured when follicle size was <1.0 cm and endometrial thickness <0.7 cm by ultrasound. Follicular fluid samples from nine patients with PCOS and nine controls were collected for proteomic analysis. Gonadotropin-releasing hormone antagonist was used for ovarian stimulation. Follicular fluid was collected by transvaginal ultrasound-guided aspiration 34–36 h after human chorionic gonadotropin administration. Only clear fluid without blood or flushing medium contamination was collected. After oocyte isolation, FF samples were centrifuged at 1,000 g for 10 min at 4°C to remove cellular components and debris. The supernatants were stored at −80°C before further processing. Each sample for the proteomics analysis was a mixture of FF from three individuals. To further validate the identified proteins, FF samples from an additional 32 patients with PCOS and 37 controls were identified by ELISA.

### Depletion of highly abundant proteins

FF samples were centrifuged at 12,000 g for 10 min at 4°C to remove cellular debris. High-abundance proteins were removed using the Proteo Miner Protein Enrichment Kit (1633006, BIO-RAD, California, United States). Protein concentrations were measured using a BCA kit (P0011, Beyotime, Jiangsu, China), according to the manufacturer’s instructions. Key reagents used in this study are listed in Supplementary Table S1.

### Trypsin digestion

An equal amount of each sample protein was used for enzymatic hydrolysis. An appropriate amount of standard protein was added to each sample, and the volumes were made equal with lysis buffer. Dithiothreitol (DTT) was added to each sample to a final concentration of 5 mM. The samples were reduced for 30 min at 56°C and then alkylated with 11 mM iodoacetamide (IAA) at room temperature for 15 min in the dark. The samples were diluted with100 mM tetraethylammonium tetra hydroborate (TEAB) to a urea concentration of less than 2 M. Trypsin was added at 1:50 (trypsin: protein) overnight for the first digestion and at 1:100 (trypsin: protein) for the second digestion lasting 4 h.

### TMT-labeling

After trypsinization, the peptides were desalted using a Strata X C18 SPE column (Phenomenex, California, United States) and vacuum-dried. The peptides were reconstituted with 0.5 M TEAB and labeled using the TMT kit (90064CH, Thermo Fisher, Massachusetts, United States). Briefly, reconstituted the thawed TMT reagent in acetonitrile. The peptide mixtures were incubated for 2 hours at room temperature, and then the samples were pooled, desalted, and dried by vacuum centrifugation.

### HPLC fractionation

The tryptic peptides were fractionated by high-pH reverse-phase HPLC using an Agilent 300Extend C18 column (250-mm length, 5-μm particles, 10-mm ID).

### Liquid chromatography-mass spectrometry analysis

The peptides were dissolved in 0.1% formic acid (solvent A) and then separated using an EASY-nLC 1,200 ultra-high performance liquid system. The liquid gradient was as follows: 0–26 min, 6–25% solvent B (0.1% formic acid in 90% acetonitrile); 26–34 min, 25–35% solvent B; 34–37 min, 35–80% solvent B; 37–40 min, 80% solvent B. The separated peptides were injected into the nano-electrospray ion source for ionization and analyzed by HF-X mass spectrometry. The electrospray voltage was 2.0 kV. The m/z scan range was 400–1,600 for a full scan, and intact peptides were detected in the Orbitrap at a resolution of 120,000. Data acquisition was performed using the data-dependent scanning (DDA) program. Peptides were selected for MS/MS, and the fragments were detected in the Orbitrap at a resolution of 15,000. The automatic gain control (AGC) was set to 5E4. The mass spectrometry proteomics data were deposited into the ProteomeXchange Consortium via the PRIDE (Perez-Riverol et al., 2022) partner repository with the dataset identifier PXD031996.

### Database search

The Maxquant search engine (v.1.5.2.8) was used to search the MS/MS data. The human UniProt database was used to search for tandem mass spectra. The FDR was adjusted to less than 1%, and the minimum score for the modified peptides was set to greater than 40. DEPs had a cut-off value of >1.3-fold or <0.769-fold change.

### Bioinformatics analysis

We derived the Gene Ontology (GO) annotation proteome from the UniProt-GOA database (http://www.ebi.ac.uk/GOA/). If the UniProt-GOA database did not annotate some identified proteins, we applied InterProScan software to analyze the GO functions of the annotated proteins using the protein sequence alignment method (Dimmer et al., 2012). Proteins were classified into three categories by the GO annotations: biological process, cellular components, and molecular functions. To further understand the DEPs, the functional enrichment analyses of the GO functions, KEGG pathways, and protein domains were performed. We used a two-tailed Fisher’s exact test to evaluate the enrichment of the DEPs with a *p*-value < 0.05. Cluster analysis was performed using Cluster 3.0 software (de Hoon et al., 2004). The protein-protein interaction networks were acquired using the STRING database (Szklarczyk et al., 2011). The interaction network was visualized using the R package “networkD3”.

### ELISA validation

Differential levels of two DEPs were verified by ELISA. Sixty-nine FF samples were collected, including 37 from PCOS patients and 32 from control patients. Two commercial ELISA kits were used to measure proteins according to the manufacturer’s instructions. One kit measured human insulin-like growth factor binding protein 1 (IGFBP-1) (DGB100, R&D, Minneapolis, United States). The intra-assay coefficient of variation (CV) changed from 4.1 to 5.6%, and the inter-assay variation changed from 6.3 to 9.5%, respectively. The second kit measured human apolipoprotein C-II (APOC2) (ELH-APOC2, Raybiotech, Norcross, GA, United States). The intra-assay and inter-assay variations were <10% and <12%, respectively.

### Statistical analysis

Statistical analysis was performed using SPSS software version 26 (IBM Corp., Armonk, NY, United States) and GraphPad Prism 8 software (GraphPad Software, San Diego, California). Continuous variables were expressed as the mean ± standard deviation. Differences between groups were compared using the two-tailed Student’s t-test. Variables with skewed distribution were presented as the median (inter-quartile range) and compared using a nonparametric test. Correlations between different variables were determined using Pearson correlation analysis.

## Results

### Clinical baseline characteristics of patients with or without PCOS

We compared the clinical baseline characteristics of nine PCOS and nine control patients. As shown in Table 1, there were no statistically significant differences in age or BMI between the two groups. Luteinizing hormone (LH) levels, the LH/FSH ratio, dehydroepiandrosterone (DHE-s), testosterone (T), and AMH levels, and the antral follicle count (AFC) were higher in women with PCOS.

**TABLE 1:** Clinical baseline characteristics of patients with and without PCOS.

Characteristic	Control (N = 9)	PCOS (N = 9)	*p*-value
Age (year)	28.89 ± 3.14	29.33 ± 2.65	0.750
BMI (kg/m^2^)	22.33 ± 1.63	23.16 ± 1.24	0.230
AFC (n)	6 (4, 7)	12 (11, 13)	0.002^a^
FSH (mIU/ml)	5.59 ± 1.29	5.88 ± 1.27	0.621
LH (mIU/ml)	5.07 ± 1.39	12.63 ± 5.76	0.004^a^
Estradiol (pg/ml)	39.59 ± 11.81	47.64 ± 23.74	0.355
Progestin (ng/ml)	0.16 (0.07, 0.31)	0.2 (0.17, 0.26)	0.327
LH/FSH ratio	0.8 (0.67, 1.01)	2.16 (1.29, 2.65)	0.004^a^
Testosterone (ng/ml)	24.13 ± 6.77	44.75 ± 18.12	0.009^a^
DHE-s (μg/dl)	212.95 ± 67.43	314.94 ± 104.58	0.021^a^
HOMA-IR	2.63 ± 1.28	4.16 ± 2.32	0.089
Prolactin (ng/ml)	14.78 ± 3.71	13.42 ± 5.9	0.551
TSH (uIU/ml)	1.96 ± 0.59	1.92 ± 0.93	0.903
AMH (ng/ml)	3.77 ± 1.18	10.57 ± 3.23	<0.001^a^
Number of follicles with diameter ≥14 mm on the day of hCG administration	10.67 ± 3.94	12.56 ± 6.42	0.463
No. of oocytes retrieved	14.11 ± 5.80	15.33 ± 6.08	0.668
No. of fertilized oocytes with 2 PN	9.56 ± 3.68	7.67 ± 3.24	0.265
No. of good-quality embryos on day 3	8.89 ± 3.62	7.44 ± 3.00	0.371

Note: Data are presented as mean ± standard deviation or median (interquartile range) for continuous variables.

BMI, body mass index; AFC, antral follicle count; FSH, follicle-stimulating hormone; LH, luteinizing hormone; DHEA-s, dehydroepiandrosterone; HOMA-IR, homeostasis model assessment of insulin resistance; TSH, Thyrotropin-releasing hormone; AMH, anti-Müllerian hormone.

a *p*-value ≤0.05 was considered statistically significant.

### Identification of DEPs

**FIGURE 1:**
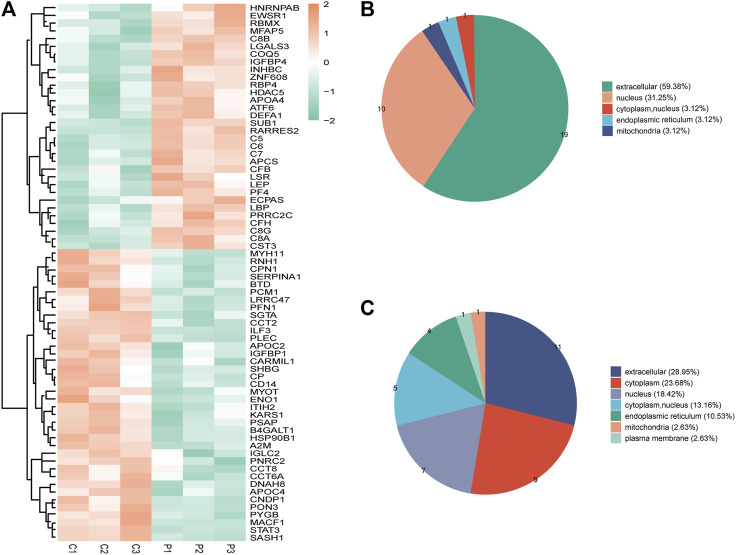
**(A)** Clustering heat map of the protein expression patterns of different groups. Red color indicates higher expression, while green indicates lower expression. Proteins without significantly differential expression were shown in white. **(B)** Distribution of the subcellular lacalization of downregulated expressed proteins for PCOS vs control **(C)** Distribution of the subcellular localization of upregulated proteins for PCOS vs control.

Seventy DEPs were identified in this study (32 upregulated and 38 downregulated) in the PCOS group. The annotations and the quantitative information for the DEPs are shown in Supplementary Table S2. Unsupervised hierarchical clustering heat maps were generated to display the DEPs (Figure 1A). Subcellular prediction was used to characterize the subcellular localization of these DEPs. Most of the upregulated proteins were localized to the extracellular space (59.38%) and nucleus (31.25%) (Figure 1B), whereas the downregulated proteins were distributed between the extracellular (28.95%), cytoplasm (23.68%), nucleus (18.42%) (Figure 1C).

### GO enrichment analysis

**FIGURE 2:**
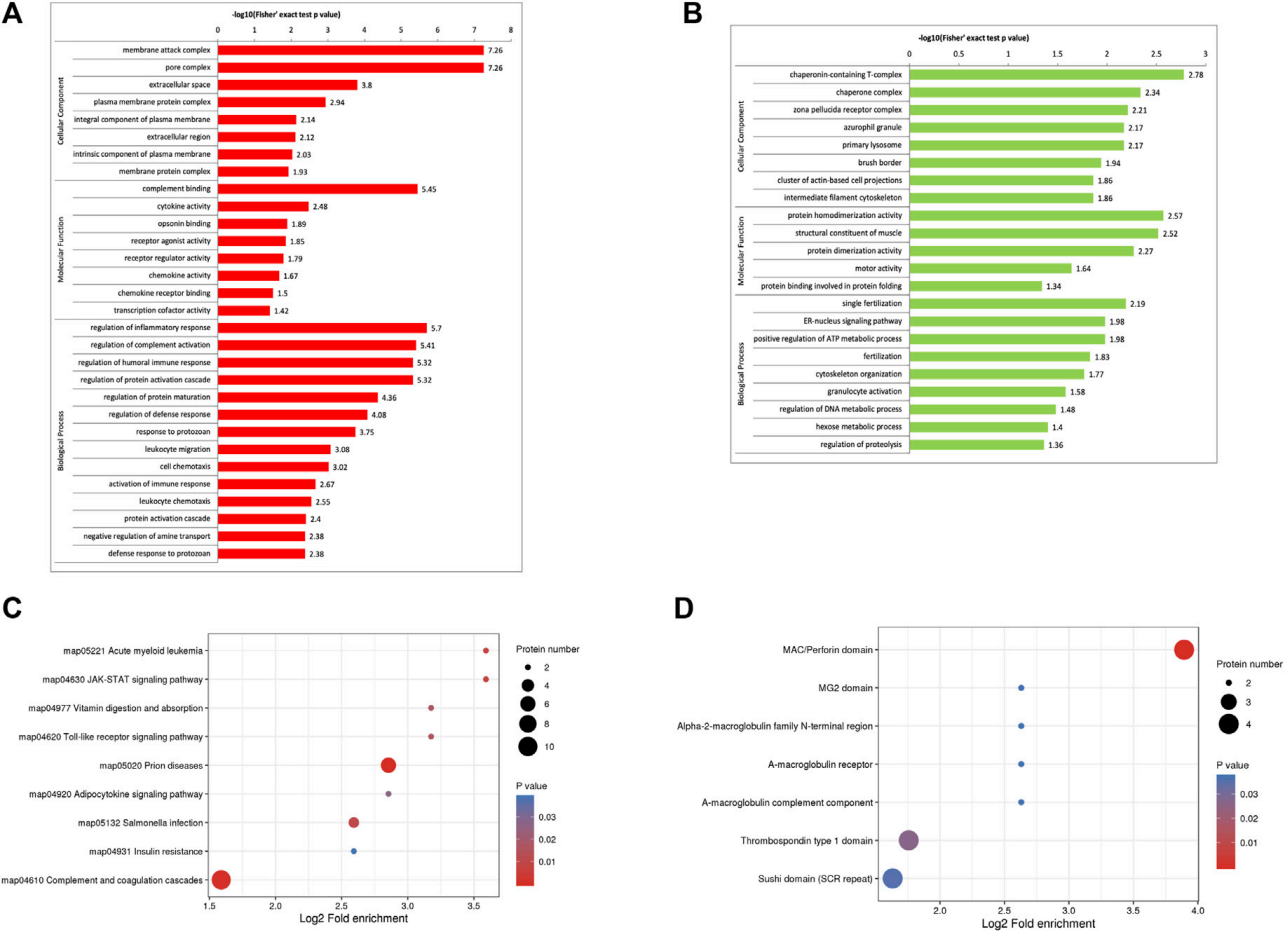
Functional enrichment of differentially expressed proteins in follicular fluid from patients with and without PCOS. **(A)** GO analysis of upregulated proteins **(B)** GO analysis of downregulated proteins. **(C)** KEGG pathway analysis **(D)** Protein domain enrichment analysis.

The GO enrichment analysis demonstrated that most of the upregulated proteins in the biological process classification were involved in regulating the inflammatory response, complement activation, protein activation cascade, humoral immune response, activation of the immune response, and leukocyte chemotaxis. Many of the upregulated proteins were associated with complement binding and cytokine activity in the molecular function classification. For the cellular components, the upregulated proteins were observed in the membrane attack complex, pore complex, and extracellular space (Figure 2A). The downregulated proteins were enriched for the ER-nucleus signaling pathway, positive regulation of the ATP metabolic process, and fertilization (Figure 2B).

### KEGG pathway and protein domain enrichment analysis

KEGG pathway enrichment analysis demonstrated that the DEPs were mainly enriched in complement and coagulation cascades, insulin resistance, and the JAK-STAT signaling pathway (Figure 2C). The domain enrichment analysis showed the DEPs mainly involved in the MAC/perforin domain, thrombospondin type 1 domain, and Sushi domain (SCR repeat) (Figure 2D).

### Clustering analysis

**FIGURE 3:**
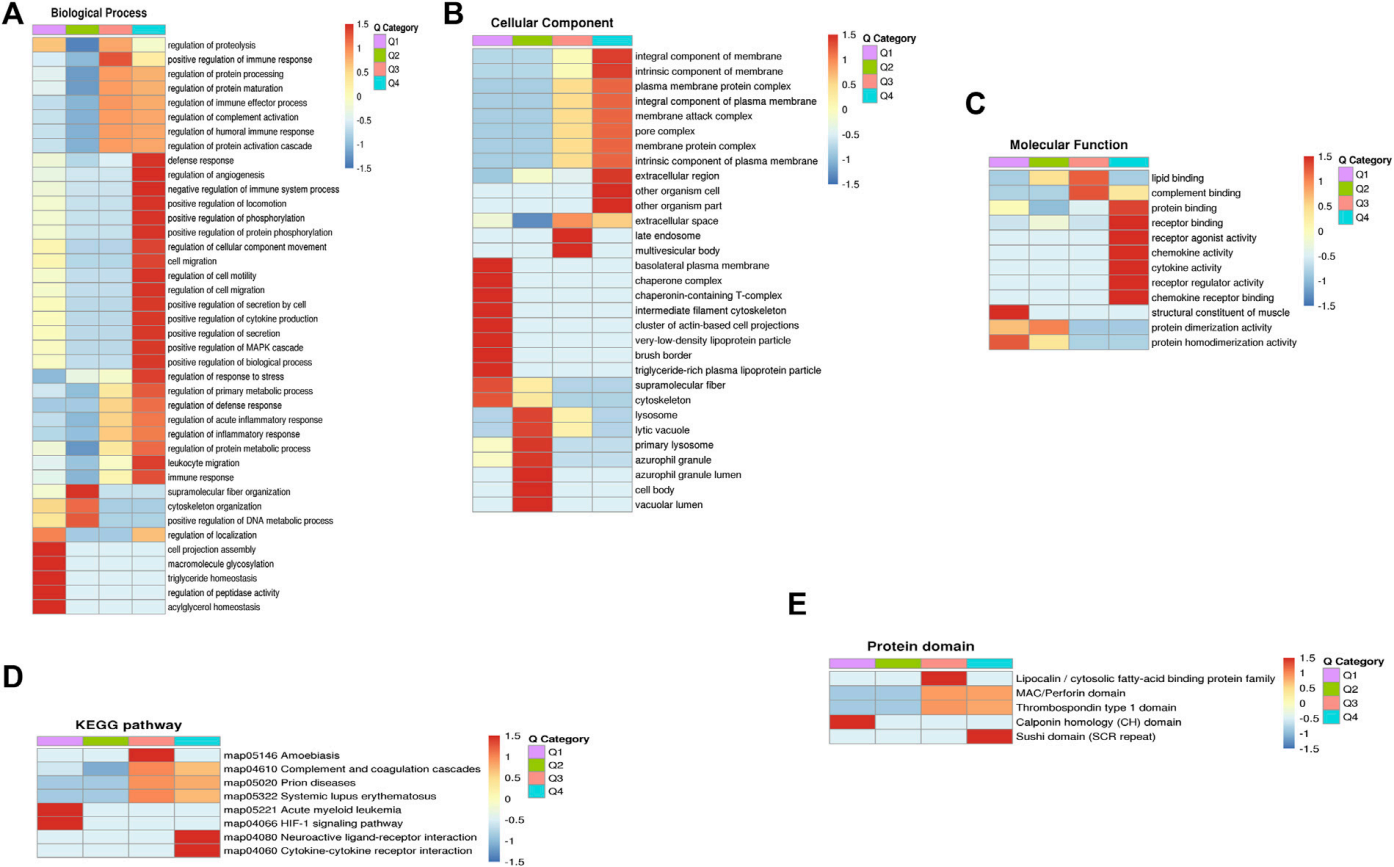
Functional enrichment-based clustering analyses of dofferentially expressed proteins in follicular fluid of PCOS patients and controls. **(A)** Biological process analysis **(B)** Cellular component analysis. **(C)** Molecular function analysis **(D)** KEGG pathway analysis **(E)** Protein domain analysis.

For the clustering analysis, the 70 DEPs were divided into four quantitative categories: Q1 (0 < P/C ratio <0.667, *p*-value < 0.05), Q2 (0.667 ≤ P/C ratio ≤0.769, *p*-value < 0.05), Q3 (1.3 ≤ P/C ratio ≤1.5, *p*-value <0.05) and Q4 (P/C ratio> 1.5, *p*-value <0.05). Q1 and Q2 represent the downregulated proteins, containing 26 and 12 proteins, respectively. Q3 and Q4 represent the upregulated proteins, containing 15 and 17 proteins, respectively. GO enrichment-based clustering analysis demonstrated that, in the biological process category, the DEPs were involved in the regulation of complement activation, the inflammatory response, the immune response, protein metabolic process, and angiogenesis (Figure 3A). The enrichment analysis for the cellular component category indicated that the DEPs were associated with the extracellular space and membrane attack complex (Figure 3B). The DEPs were mainly associated with lipid binding, complement binding, cytokine activity, and chemokine activity in the molecular function category (Figure 3C). The KEGG-based enrichment analysis indicated that complement and coagulation cascades and cytokine-cytokine receptor interaction were associated with PCOS development (Figure 3D). Based on the domain enrichment-based clustering analysis, the DEPs were clustered with the lipocalin/cytosolic fatty-acid binding protein family and the MAC/Perforin domain (Figure 3E).

### Protein-protein interaction network analysis

**FIGURE 4:**
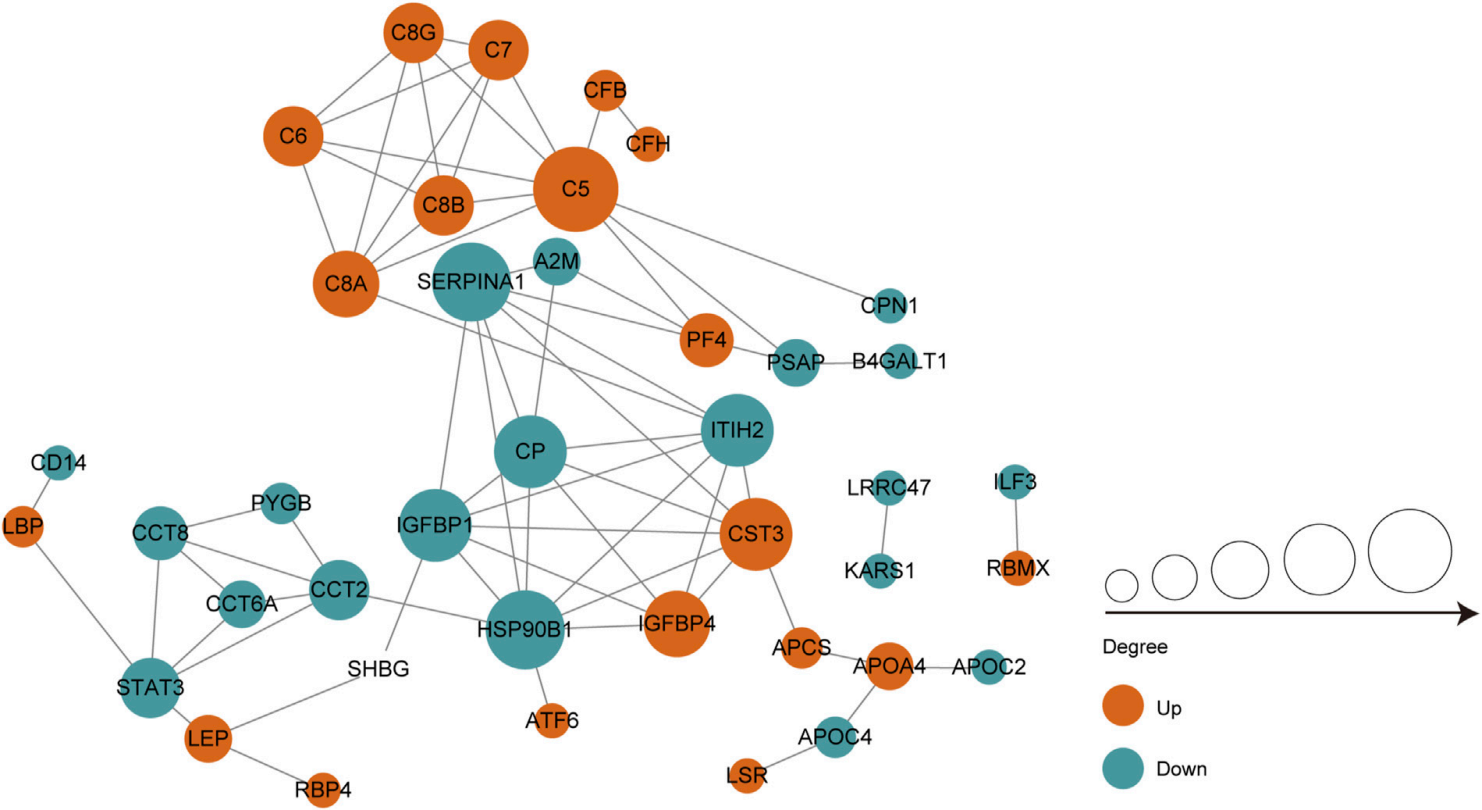
PPI co-expression network of differentially expressed proteins (DEPs) in follicular fluid of PCOS women and controls. Forty of the 70 DEPs were predicted to participate in direct PPIs. Nodes represent proteins and lines represent PPIs. The degree determines the node size, where red represents upregulated and the green represents downregulated.

We performed protein-protein interaction (PPI) proteomics network analysis using STRING and Cytoscape to better understand potential PPIs for the DEPs and related intact proteins. Forty DEPs exhibited direct interactions, including 19 upregulated proteins and 21 downregulated proteins (Figure 4). The DEPs were presented as nodes, with the upregulated and downregulated proteins illustrated in red and green, respectively. Potential interactions between the DEPs are indicated by lines. Notably, the DEPs were mainly associated with the complement and coagulation cascades, inflammation, and immunity. Moreover, many of the upregulated proteins belonged to the complement and coagulation cascades, such as complement C5 (C5), C6, and C7.

### Validation of selected DEPs

**FIGURE 5:**
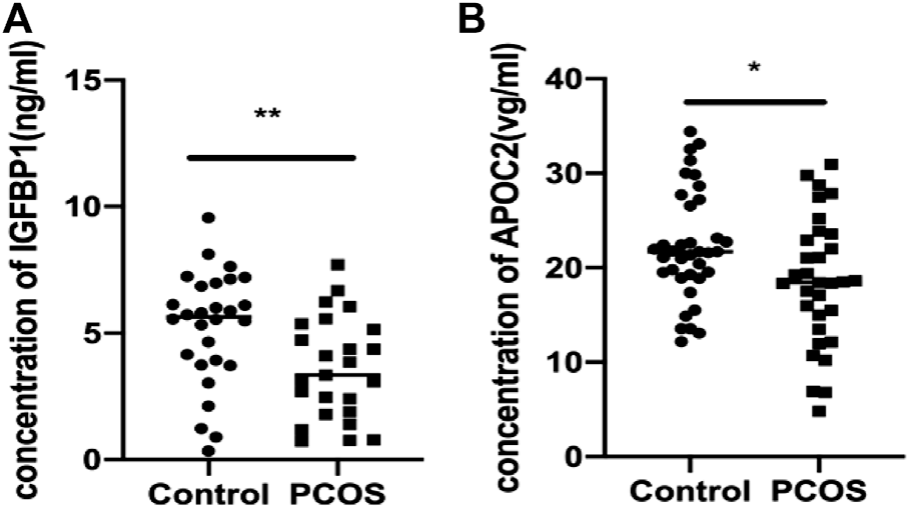
Validation of IGFBP1 and APOC2 concentrations in follicular fluid of PCOS patients and the controls using ELISA. Graphical results are shown in mean ± SD, *p < 0.05, **p < 0.01, ***p < 0.001. **(A)** Insulin-like Growth Factor Binding protein 1(IGFBP1_. **(B)** Apolipoprotein C2 (APOC2).

ELISA was used to validate the differential abundance of two DEPs (IGFBP and APOC2). Sixty-nine FF samples were collected, including 37 from PCOS patients and 32 from control patients. Consistent with the proteomic analysis, the FF levels of IGFBP and APOC2 in the PCOS group were significantly lower than those in the control group (Figures 5A,B).

### The correlation of follicular IGFBP1 and APOC2 with the clinical characteristics of patients

**FIGURE 6:**
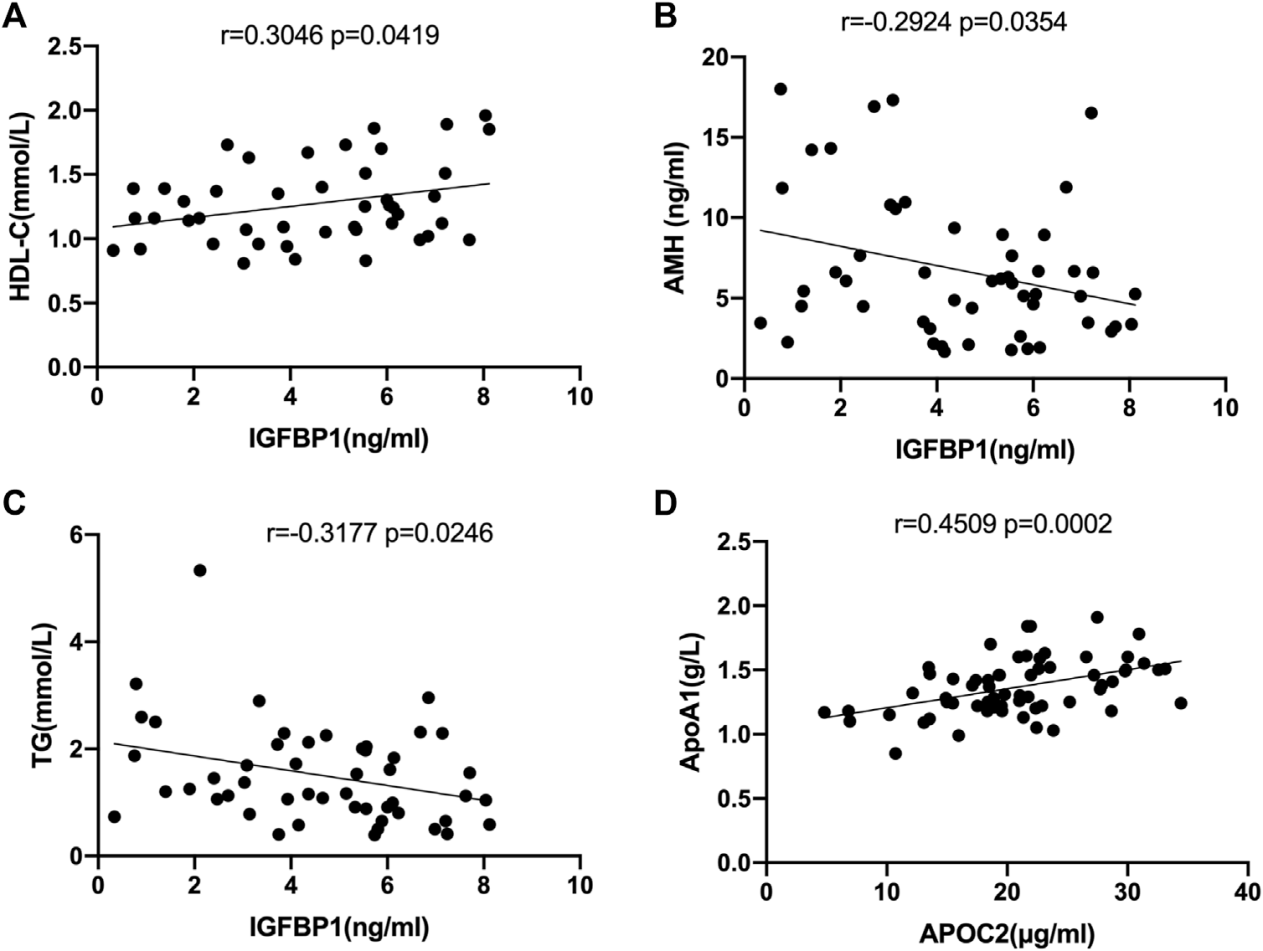
Correlation analysis of selected DEPs with clinical characteristics in all follicular fluid samples. **(A)** Correlation analysis of IGFPB1 with LDL-C (r = 0.3046, *p* = 0.0419). **(B)**Correlation analysis of IGFPB1 with AMH (r = 0.2924, *p* = 0.0354). **(C)** Correlation analysis of IGFPB1 with TG (r = -0.3177, *p* = 0.0246). **(D)** Correlation analysis of APOC2 with APOA1 (r = 0.4509, *p* = 0.0002).

Correlation analysis between the levels of IGFBP1 and APOC2 in FF and the clinical data of the recruited patients revealed that IGFBP1 was positively correlated with high-density lipoprotein cholesterol (r = 0.3046, *p* = 0.0419, Figure 6A), but negatively correlated with AMH (r = −0.2924, *p* = 0.0354, Figure 6B) and triglycerides (r = −0.3177, *p* = 0.0246, Figure 6C). APOC2 was negatively correlated with apolipoprotein A1(APOA1) (r = 0.4509, *p* = 0.0002, Figure 6D).

## Discussion

In this study, a TMT-based quantitative proteomic analysis tool was employed to investigate the protein profiles in the FF of women with or without PCOS. We identified 70 DEPs (32 upregulated, 38 downregulated). GO terms and significant pathways associated with the DEPs were also identified, including regulation of the inflammatory response, complement and coagulation cascades, activation of the immune response, lipid transport, regulation of the protein metabolic process, insulin resistance, and adipocytokine signaling pathway, which were dysregulated in the FF of women with PCOS.

PCOS is a state of chronic low-grade inflammation associated with autoimmune disease (Rudnicka et al., 2021). In our study, some upregulated proteins in PCOS patients were enriched in the complement coagulation cascade, inflammatory response, and immune response. The complement system consists of a group of proteins that rapidly activate in a cascade to orchestrate inflammatory and immune responses in organisms. The complement cascade is activated by three main complement activation pathways—the classical pathway, the lectin pathway, and the alternative pathway (Holers, 2014; Blatt et al., 2016; Reichhardt and Meri, 2018; Goldberg and Ackerman, 2020). All three pathways can activate the terminal pathway, including the formation of the membrane attack complex (MAC) (Ort et al., 2020), which is a multi-protein pore composed of C5B, C6, C7, C8A, C8B, and C9 (Hadders et al., 2007; Rosado et al., 2007; Bayly-Jones et al., 2017). Previous studies have reported that some complement proteins are important components of the FF and play an important role in reproduction (Jarkovska et al., 2010; Yoo et al., 2013; Walter et al., 2019). In this study, increased levels of complement proteins were found in women with PCOS, with eight upregulated proteins clustered into a large group of complement proteins (C5, C6, C7, C8A, C8B, C8G, complement factor B, and complement factor H), indicating the distinct regulation of the complement cascade in the FF of PCOS. Moreover, the protein domain and KEGG pathway enrichment analysis showed that some complement proteins (C6, C7, C8A, and C8B) have MAC/perforin domains and are mainly associated with the complement and coagulation cascades. The links between the DEPs and the results derived from the different enrichment analyses demonstrated complement activation in the FF of patients with PCOS, which might play a role in the development of this condition.

Consistent with our study, significantly higher C5 levels have been identified in patients with PCOS (Zhang et al., 2019; Lewis et al., 2021). However, there have been no studies on C6 and C7 in the FF of patients with PCOS; their effect on PCOS pathogenesis remains to be investigated. We also detected that some upregulated DEPs were enriched in the inflammatory pathway, such as the elevation of lipopolysaccharide-binding protein (LBP). LBP is one of the most important ligands for lipopolysaccharide (LPS), which can transfer LPS to the LPS receptor, leading to an active inflammatory response (Fenton and Golenbock, 1998). A recent study showed that serum LBP levels are significantly increased in PCOS compared to BMI-matched controls (Zhu et al., 2016).

Alterations in the expression of proteins involved in lipid transport and metabolism have also been observed in PCOS. Dysregulation of lipoproteins in women with PCOS has been demonstrated and could be a biomarker for long-term adverse health outcomes (Wild et al., 2011; Wild, 2012). Furthermore, many apolipoproteins have been identified in FF and may have an essential impact on reproduction (Von Wald et al., 2010). To our knowledge, altered APOC2 levels in the FF of patients with PCOS have not been previously reported. We demonstrated significantly decreased APOC2 levels in this study and validated these findings in the FF of PCOS patients. APOC2 is known for its role in lipid metabolism, acting as a physiological activator of lipoprotein lipase (LPL) (Schuster et al., 2011). APOC2 is a vital component of low-density, very low-density, and high-density lipoproteins. APOC2 deficiency can cause severe hypertriglyceridemia and lead to cardiovascular disease (Yang et al., 2020). In addition, we found that APOC2 was positively correlated with APOA1. APOA1 was significantly reduced in PCOS patients independent of BMI or hyperandrogenism (Wild, 2012). Further studies are necessary to understand the exact mechanism of action for APOC2 in the pathogenesis of PCOS.

We also found that APOA4 was increased in the FF of patients with PCOS, consistent with a previous study (Kim et al., 2013). APOA4 is a lipid-binding protein that has been associated with lipid transport, lipid metabolism, and metabolic regulation (Green et al., 1980; Araki et al., 2004). We found higher levels of retinol-binding protein 4 (RBP4) in PCOS, an adipokine that can impair insulin sensitivity throughout the body (Muoio and Newgard, 2005). Several studies have shown that interventions to improve insulin sensitivity can lower serum RBP4 levels (Balagopal et al., 2007; Haider et al., 2007). We also identified and validated that IGFBP1 was decreased in the FF of PCOS. Previous studies reported decreased serum IGFBP1 levels in patients with PCOS (Conway et al., 1990; van Dessel et al., 1999). However, fewer studies have evaluated follicular IGFBP1 levels in patients with PCOS. IGFBP1 belongs to a family of IGFBPs, which regulate the bioavailability of insulin-like growth factor-I (IGF-I) (Firth and Baxter, 2002). IGFBP1 in the FF was secreted by granulosa cells and diffused by liver circulation (Suikkari et al., 1991). IGFBP1 production is inhibited by insulin (Leroith et al., 1995; Dunaif, 1997). It is well known that women with PCOS likely have insulin resistance and hyperinsulinemia, independent of obesity (Diamanti-Kandarakis and Dunaif, 2012). Therefore, insulin resistance in patients with PCOS might inhibit the production of IGFBP1. In our study, we also found that follicular IGFBP1 levels were negatively correlated with the serum levels of AMH and triglycerides and positively correlated with the HDL-C levels. AMH is often used as an indicator of ovarian reserve (Tal et al., 2014), which is increased in PCOS and associated with various reproductive and metabolic alterations (Garg and Tal, 2016). Women with PCOS usually have higher triglyceride levels but lower HDL-C levels (Wild, 2012).

In our study, relatively more DEPs were identified. A total of 70 DEPs were identified in the PCOS group. Furthermore, we identified new DEPs in the FF of PCOS that were never reported, such as APOC2, C6, and C7. Several of the DEPs identified in our study were previously reported, such as APOA4 (Kim et al., 2013) and FN1 (Ambekar et al., 2015). While we have also identified some that have not been reported previously. To date, proteins identified through proteomic studies of PCOS have been involved in a variety of biological pathways and physiological processes. Some of the biological pathways and physiological processes we have identified in our study were consistent with previous studies (Insenser and Escobar-Morreale, 2013; Ambekar et al., 2015; Zhang et al., 2019), while we have also identified some that have not been reported previously. Our results complement the identified DEPs in PCOS found in previous studies and contribute to a better understanding of the pathophysiological mechanisms of PCOS.

This study had several limitations. First, due to the relatively small number of patients included in our study, further research is needed with a larger number of patients to confirm these findings. Second, there was no direct functional evidence demonstrating whether these DEPs could contribute to the underlying mechanism for the pathogenesis of PCOS. All of our findings were preliminary; further research is needed.

## Conclusion

We employed TMT-based proteomics combined with bioinformatics analysis to identify DEPs involved in PCOS. The DEPs were associated with inflammatory, immunological, metabolic, and lipid transport alterations and may play a role in the development of PCOS. These findings improved our understanding of the pathogenic mechanisms underlying PCOS. The functions of these proteins in PCOS pathogenesis require further study.

## Data Availability

The datasets presented in this study can be found in online repositories. The names of the repository/repositories and accession number(s) can be found below: https://www.ebi.ac.uk/pride/archive/, PXD031996.
