# Supplementary material for: Proteomics and bioinformatics analysis of follicular fluid from patients with polycystic ovary syndrome
Source: Front Mol Biosci. 2022 Aug 22;9:956406. doi: 10.3389/fmolb.2022.956406 (PMC9441494; doi:10.3389/fmolb.2022.956406)
Supplement: Supplementary file 1 [file Table1.docx]

Table S1. Tabular presentation of important reagents and materials used for this study

| Reagents and Materials | Company | Catalog number |
| --- | --- | --- |
| Trypsin | Promega | SRT200601 |
| Proteo Miner Protein Enrichment Kit | BIO-RAD | 1633006 |
| Acetonitrile | Fisher Chemical | 197164 |
| Iodoacetamide (IAA) | Sigma-Aldrich | WXBB6825V |
| Dithiothreitol (DTT) | Sigma-Aldrich | SLBW6181 |
| Tetraethylammonium borohydride (TEAB) | Sigma-Aldrich | BCCD9826 |
| TMT kit | ThermoFisher Scientific | 90064CH |
| BCA kit | Beyotime | P0011 |
