# Supplementary material for: Proteomics and bioinformatics analysis of follicular fluid from patients with polycystic ovary syndrome
Source: Front Mol Biosci. 2022 Aug 22;9:956406. doi: 10.3389/fmolb.2022.956406 (PMC9441494; doi:10.3389/fmolb.2022.956406)
Supplement: Supplementary file 2 [file Table2.docx]

Table S2 List of differentially expressed proteins identified in the follicular fluid from patients with PCOS vs. controls.

| **Protein accession** | **Protein description** | **Gene name** | **PCOS/Control Ratio** | **PCOS/Control**  **P value** | **Regulated Type** |
| --- | --- | --- | --- | --- | --- |
| P38159 | RNA-binding motif protein, X chromosome OS=Homo sapiens OX=9606 GN=RBMX PE=1 SV=3 | RBMX | 2.21 | 0.007147553 | Up |
| P13671 | Complement component C6 OS=Homo sapiens OX=9606 GN=C6 PE=1 SV=3 | C6 | 1.995 | 0.002735396 | Up |
| P18850 | Cyclic AMP-dependent transcription factor ATF-6 alpha OS=Homo sapiens OX=9606 GN=ATF6 PE=1 SV=3 | ATF6 | 1.885 | 0.019168127 | Up |
| P02776 | Platelet factor 4 OS=Homo sapiens OX=9606 GN=PF4 PE=1 SV=2 | PF4 | 1.833 | 0.008554582 | Up |
| Q9UQL6 | Histone deacetylase 5 OS=Homo sapiens OX=9606 GN=HDAC5 PE=1 SV=2 | HDAC5 | 1.726 | 0.049620794 | Up |
| Q01844 | RNA-binding protein EWS OS=Homo sapiens OX=9606 GN=EWSR1 PE=1 SV=1 | EWSR1 | 1.712 | 0.046649323 | Up |
| P10643 | Complement component C7 OS=Homo sapiens OX=9606 GN=C7 PE=1 SV=2 | C7 | 1.694 | 0.015052374 | Up |
| P22692 | Insulin-like growth factor-binding protein 4 OS=Homo sapiens OX=9606 GN=IGFBP4 PE=1 SV=2 | IGFBP4 | 1.692 | 0.002547938 | Up |
| P01031 | Complement C5 OS=Homo sapiens OX=9606 GN=C5 PE=1 SV=4 | C5 | 1.683 | 0.002308219 | Up |
| P06727 | Apolipoprotein A-IV OS=Homo sapiens OX=9606 GN=APOA4 PE=1 SV=3 | APOA4 | 1.683 | 0.044631461 | Up |
| Q13361 | Microfibrillar-associated protein 5 OS=Homo sapiens OX=9606 GN=MFAP5 PE=1 SV=1 | MFAP5 | 1.645 | 0.0217613 | Up |
| P00751 | Complement factor B OS=Homo sapiens OX=9606 GN=CFB PE=1 SV=2 | CFB | 1.619 | 0.036327644 | Up |
| P41159 | Leptin OS=Homo sapiens OX=9606 GN=LEP PE=1 SV=1 | LEP | 1.618 | 0.022891497 | Up |
| Q99969 | Retinoic acid receptor responder protein 2 OS=Homo sapiens OX=9606 GN=RARRES2 PE=1 SV=1 | RARRES2 | 1.565 | 0.001050955 | Up |
| Q9Y520 | Protein PRRC2C OS=Homo sapiens OX=9606 GN=PRRC2C PE=1 SV=4 | PRRC2C | 1.563 | 0.029532688 | Up |
| P17931 | Galectin-3 OS=Homo sapiens OX=9606 GN=LGALS3 PE=1 SV=5 | LGALS3 | 1.56 | 0.012296534 | Up |
| P02743 | Serum amyloid P-component OS=Homo sapiens OX=9606 GN=APCS PE=1 SV=2 | APCS | 1.512 | 0.01414741 | Up |
| Q5VYK3 | Proteasome adapter and scaffold protein ECM29 OS=Homo sapiens OX=9606 GN=ECPAS PE=1 SV=2 | ECPAS | 1.495 | 0.02627681 | Up |
| Q99729 | Heterogeneous nuclear ribonucleoprotein A/B OS=Homo sapiens OX=9606 GN=HNRNPAB PE=1 SV=2 | HNRNPAB | 1.464 | 0.03785878 | Up |
| P07360 | Complement component C8 gamma chain OS=Homo sapiens OX=9606 GN=C8G PE=1 SV=3 | C8G | 1.424 | 0.003157672 | Up |
| P59665 | Neutrophil defensin 1 OS=Homo sapiens OX=9606 GN=DEFA1 PE=1 SV=1 | DEFA1 | 1.403 | 0.015941927 | Up |
| P55103 | Inhibin beta C chain OS=Homo sapiens OX=9606 GN=INHBC PE=2 SV=1 | INHBC | 1.403 | 0.01737049 | Up |
| P01034 | Cystatin-C OS=Homo sapiens OX=9606 GN=CST3 PE=1 SV=1 | CST3 | 1.399 | 0.005046687 | Up |
| P07358 | Complement component C8 beta chain OS=Homo sapiens OX=9606 GN=C8B PE=1 SV=3 | C8B | 1.387 | 0.009648401 | Up |
| P07357 | Complement component C8 alpha chain OS=Homo sapiens OX=9606 GN=C8A PE=1 SV=2 | C8A | 1.382 | 0.00742529 | Up |
| P53999 | Activated RNA polymerase II transcriptional coactivator p15 OS=Homo sapiens OX=9606 GN=SUB1 PE=1 SV=3 | SUB1 | 1.368 | 0.00064953 | Up |
| P08603 | Complement factor H OS=Homo sapiens OX=9606 GN=CFH PE=1 SV=4 | CFH | 1.354 | 0.018629807 | Up |
| P18428 | Lipopolysaccharide-binding protein OS=Homo sapiens OX=9606 GN=LBP PE=1 SV=3 | LBP | 1.351 | 0.00624194 | Up |
| P02753 | Retinol-binding protein 4 OS=Homo sapiens OX=9606 GN=RBP4 PE=1 SV=3 | RBP4 | 1.33 | 0.017864814 | Up |
| Q86X29 | Lipolysis-stimulated lipoprotein receptor OS=Homo sapiens OX=9606 GN=LSR PE=1 SV=4 | LSR | 1.321 | 0.044513858 | Up |
| Q9ULD9 | Zinc finger protein 608 OS=Homo sapiens OX=9606 GN=ZNF608 PE=1 SV=4 | ZNF608 | 1.313 | 0.041100053 | Up |
| Q5HYK3 | 2-methoxy-6-polyprenyl-1,4-benzoquinol methylase, mitochondrial OS=Homo sapiens OX=9606 GN=COQ5 PE=1 SV=2 | COQ5 | 1.301 | 0.004578648 | Up |
| P07737 | Profilin-1 OS=Homo sapiens OX=9606 GN=PFN1 PE=1 SV=2 | PFN1 | 0.763 | 0.019208188 | Down |
| Q9NPJ4 | Proline-rich nuclear receptor coactivator 2 OS=Homo sapiens OX=9606 GN=PNRC2 PE=1 SV=1 | PNRC2 | 0.762 | 0.031656957 | Down |
| P78371 | T-complex protein 1 subunit beta OS=Homo sapiens OX=9606 GN=CCT2 PE=1 SV=4 | CCT2 | 0.762 | 0.003571584 | Down |
| P13489 | Ribonuclease inhibitor OS=Homo sapiens OX=9606 GN=RNH1 PE=1 SV=2 | RNH1 | 0.758 | 0.005994156 | Down |
| P14625 | Endoplasmin OS=Homo sapiens OX=9606 GN=HSP90B1 PE=1 SV=1 | HSP90B1 | 0.752 | 0.005574827 | Down |
| Q8N1G4 | Leucine-rich repeat-containing protein 47 OS=Homo sapiens OX=9606 GN=LRRC47 PE=1 SV=1 | LRRC47 | 0.748 | 0.0113645 | Down |
| P35749 | Myosin-11 OS=Homo sapiens OX=9606 GN=MYH11 PE=1 SV=3 | MYH11 | 0.736 | 0.011773786 | Down |
| P07602 | Prosaposin OS=Homo sapiens OX=9606 GN=PSAP PE=1 SV=2 | PSAP | 0.721 | 0.010603639 | Down |
| P43251 | Biotinidase OS=Homo sapiens OX=9606 GN=BTD PE=1 SV=2 | BTD | 0.708 | 0.036711422 | Down |
| O43765 | Small glutamine-rich tetratricopeptide repeat-containing protein alpha OS=Homo sapiens OX=9606 GN=SGTA PE=1 SV=1 | SGTA | 0.707 | 0.007977015 | Down |
| P15169 | Carboxypeptidase N catalytic chain OS=Homo sapiens OX=9606 GN=CPN1 PE=1 SV=1 | CPN1 | 0.683 | 0.031204923 | Down |
| P11216 | Glycogen phosphorylase, brain form OS=Homo sapiens OX=9606 GN=PYGB PE=1 SV=5 | PYGB | 0.682 | 0.012948501 | Down |
| P50990 | T-complex protein 1 subunit theta OS=Homo sapiens OX=9606 GN=CCT8 PE=1 SV=4 | CCT8 | 0.66 | 0.049462653 | Down |
| P08833 | Insulin-like growth factor-binding protein 1 OS=Homo sapiens OX=9606 GN=IGFBP1 PE=1 SV=1 | IGFBP1 | 0.652 | 0.024133499 | Down |
| Q9UBF9 | Myotilin OS=Homo sapiens OX=9606 GN=MYOT PE=1 SV=2 | MYOT | 0.651 | 0.020332906 | Down |
| Q15154 | Pericentriolar material 1 protein OS=Homo sapiens OX=9606 GN=PCM1 PE=1 SV=5 | PCM1 | 0.651 | 0.003453551 | Down |
| P19823 | Inter-alpha-trypsin inhibitor heavy chain H2 OS=Homo sapiens OX=9606 GN=ITIH2 PE=1 SV=2 | ITIH2 | 0.65 | 0.017943966 | Down |
| P02655 | Apolipoprotein C-II OS=Homo sapiens OX=9606 GN=APOC2 PE=1 SV=1 | APOC2 | 0.647 | 0.026762207 | Down |
| Q15046 | Lysine--tRNA ligase OS=Homo sapiens OX=9606 GN=KARS1 PE=1 SV=3 | KARS1 | 0.625 | 0.045405151 | Down |
| P0DOY2 | Immunoglobulin lambda constant 2 OS=Homo sapiens OX=9606 GN=IGLC2 PE=1 SV=1 | IGLC2 | 0.62 | 0.038428326 | Down |
| Q15166 | Serum paraoxonase/lactonase 3 OS=Homo sapiens OX=9606 GN=PON3 PE=1 SV=3 | PON3 | 0.618 | 0.007624954 | Down |
| Q5VZK9 | F-actin-uncapping protein LRRC16A OS=Homo sapiens OX=9606 GN=CARMIL1 PE=1 SV=1 | CARMIL1 | 0.611 | 0.033447083 | Down |
| P00450 | Ceruloplasmin OS=Homo sapiens OX=9606 GN=CP PE=1 SV=1 | CP | 0.603 | 0.029327338 | Down |
| P01009 | Alpha-1-antitrypsin OS=Homo sapiens OX=9606 GN=SERPINA1 PE=1 SV=3 | SERPINA1 | 0.602 | 0.033538689 | Down |
| Q12906 | Interleukin enhancer-binding factor 3 OS=Homo sapiens OX=9606 GN=ILF3 PE=1 SV=3 | ILF3 | 0.576 | 5.88E-06 | Down |
| P06733 | Alpha-enolase OS=Homo sapiens OX=9606 GN=ENO1 PE=1 SV=2 | ENO1 | 0.572 | 0.041253343 | Down |
| P40227 | T-complex protein 1 subunit zeta OS=Homo sapiens OX=9606 GN=CCT6A PE=1 SV=3 | CCT6A | 0.562 | 0.031080475 | Down |
| P55056 | Apolipoprotein C-IV OS=Homo sapiens OX=9606 GN=APOC4 PE=1 SV=1 | APOC4 | 0.547 | 0.019130742 | Down |
| P08571 | Monocyte differentiation antigen CD14 OS=Homo sapiens OX=9606 GN=CD14 PE=1 SV=2 | CD14 | 0.546 | 0.032021779 | Down |
| P01023 | Alpha-2-macroglobulin OS=Homo sapiens OX=9606 GN=A2M PE=1 SV=3 | A2M | 0.54 | 0.001254729 | Down |
| P15291 | Beta-1,4-galactosyltransferase 1 OS=Homo sapiens OX=9606 GN=B4GALT1 PE=1 SV=5 | B4GALT1 | 0.534 | 0.005234797 | Down |
| Q96JB1 | Dynein heavy chain 8, axonemal OS=Homo sapiens OX=9606 GN=DNAH8 PE=1 SV=2 | DNAH8 | 0.51 | 0.005770398 | Down |
| Q9UPN3 | Microtubule-actin cross-linking factor 1, isoforms 1/2/3/5 OS=Homo sapiens OX=9606 GN=MACF1 PE=1 SV=4 | MACF1 | 0.475 | 0.007040872 | Down |
| Q96KN2 | Beta-Ala-His dipeptidase OS=Homo sapiens OX=9606 GN=CNDP1 PE=1 SV=4 | CNDP1 | 0.463 | 0.008397559 | Down |
| O94885 | SAM and SH3 domain-containing protein 1 OS=Homo sapiens OX=9606 GN=SASH1 PE=1 SV=3 | SASH1 | 0.391 | 0.001158073 | Down |
| P40763 | Signal transducer and activator of transcription 3 OS=Homo sapiens OX=9606 GN=STAT3 PE=1 SV=2 | STAT3 | 0.328 | 0.000987912 | Down |
| P04278 | Sex hormone-binding globulin OS=Homo sapiens OX=9606 GN=SHBG PE=1 SV=2 | SHBG | 0.321 | 0.02630369 | Down |
| Q15149 | Plectin OS=Homo sapiens OX=9606 GN=PLEC PE=1 SV=3 | PLEC | 0.153 | 0.001474759 | Down |
